# Current Applications and Future Perspectives of the Use of 3D Printing in Anatomical Training and Neurosurgery

**DOI:** 10.3389/fnana.2016.00069

**Published:** 2016-06-24

**Authors:** Vivek Baskaran, Goran Štrkalj, Mirjana Štrkalj, Antonio Di Ieva

**Affiliations:** ^1^ACT HealthCanberra, ACT, Australia; ^2^Faculty of Science and Engineering, Macquarie UniversitySydney, NSW, Australia; ^3^Department of Biomedical Sciences, Faculty of Medicine and Health Sciences, Macquarie UniversitySydney, NSW, Australia; ^4^Neurosurgery Unit, Faculty of Medicine and Health Sciences, Macquarie UniversitySydney, NSW, Australia; ^5^Cancer Division, Garvan Institute of Medical ResearchSydney, NSW, Australia

**Keywords:** 3D printing, computer aided design (CAD), rapid prototyping, surgery, education, anatomy, neurosurgery

## Abstract

3D printing is a form of rapid prototyping technology, which has led to innovative new applications in biomedicine. It facilitates the production of highly accurate three dimensional objects from substrate materials. The inherent accuracy and other properties of 3D printing have allowed it to have exciting applications in anatomy education and surgery, with the specialty of neurosurgery having benefited particularly well. This article presents the findings of a literature review of the Pubmed and Web of Science databases investigating the applications of 3D printing in anatomy and surgical education, and neurosurgery. A number of applications within these fields were found, with many significantly improving the quality of anatomy and surgical education, and the practice of neurosurgery. They also offered advantages over existing approaches and practices. It is envisaged that the number of useful applications will rise in the coming years, particularly as the costs of this technology decrease and its uptake rises.

## Introduction

3D printing is an exciting advanced manufacturing technology, which has important applications in biomedicine. It is a form of rapid prototyping, which enables the creation of three-dimensional structures from computer-aided design (CAD) data sets. This is physically achieved through an additive layering process (Peltola et al., 2008; Berman, 2012). 3D printing has opened new avenues for the manufacturing of objects across a number of fields. In particular, it has shown promise and yielded applications in anatomical and surgical training and a number of surgical specialties, including neurosurgery. The applications in the field of neurosurgery have been highly innovative and have sought to improve the experience of trainees and also day-to-day neurosurgical practice. This article will first briefly discuss the technology of 3D printing and rapid prototyping and then present findings of a literature review investigating the roles and applications of 3D printing in anatomical and surgical training as well as neurosurgery.

## Methods

A literature review was conducted using the PubMed and Web of Science databases. Database search terms used included: “3D printing,” “rapid prototyping,” “surgery,” “education,” “anatomy,” “anatomical,” “neurosurgery,” and “cranioplasty.” Articles were selected for inclusion in the study based on a reading of the abstract, with the intention to include material directly relevant to the study aims. There was no restriction on the date of article publication. Articles were excluded if not written in the English language.

## Rapid prototyping and 3D printing technology

Rapid prototyping represents a group of advanced manufacturing techniques, including 3D printing machines, which have been developed over the last 20 to 30 years (Berman, 2012). They utilize computer aided design (CAD) information to produce three-dimensional objects via an additive layering process (Peltola et al., 2008; Berman, 2012). Digital Imaging and Communications in Medicine (DICOM) imaging data, such as that obtained from CT or MRI, can be incorporated into the CAD models, thereby allowing patient specific information to be translated into the three dimensional end products (Peltola et al., 2008; Rengier et al., 2010; Berman, 2012; Naftulin et al., 2015). After undergoing processing, the information is ultimately sent to a rapid prototyping machine in the industry standard STL file format (Berman, 2012; Naftulin et al., 2015).

Processing enables the user to extract the desired information from DICOM images (a process known as segmentation) in preparation for export to a rapid prototyping machine (Naftulin et al., 2015; Marro et al., 2016). An example of this is representation of brain pial surface from a source two dimensional brain MRI image, which is then subsequently exported in the STL file format to a rapid prototyping machine for production (Naftulin et al., 2015). Such processing can be achieved through a number of open source software programs, including Freesurfer and InVesalius (Naftulin et al., 2015). Further editing of STL files is also possible through 3D modeling software, such as Blender, which can enable the user to selectively crop STL file information prior to 3D model production (Naftulin et al., 2015).

Rapid prototyping machine generates 3D models by sequentially adding and fusing one layer of material onto another (Peltola et al., 2008; Rengier et al., 2010; Berman, 2012). The different types of machines can be differentiated based upon the layering method used (Berman, 2012). 3D printers use powder based materials, which are then fused layer-by-layer using a liquid adhesive hardened by an ultraviolet laser (Peltola et al., 2008; Berman, 2012). Once the final layer has been added, excess liquid adhesive can be removed via a chemical bath (Peltola et al., 2008). 3D printers work in a manner similar to inkjet printers. The powder material is deposited either via a roller or piston (Rengier et al., 2010; Berman, 2012). Selective laser sintering, which uses a laser to fuse particles of thermoplastic, metal, ceramic, or glass powders, is another powder based rapid prototyping technique (Rengier et al., 2010). The remaining techniques can be classified by use of either a solid or liquid based system, with examples of the former including fused deposition modeling and of the latter being stereolithography and two-photon polymerization (Berman, 2012). The materials used in rapid prototyping depend on the individual technique and the desired application for the end product.

The 3D printing materials used for surgical applications are typically powder based and vary depending on the nature of the application. Examples of materials include polymers, ceramics, plastics, resins, super alloys, stainless steel, and titanium (Peltola et al., 2008; Berman, 2012). The materials must also be biocompatible and durable, if being used to create a prosthetic or implant. Materials used for simulation and teaching purposes are often chosen to replicate real tissue characteristics, with multiple materials often used to accurately represent complex tissue architecture (Waran et al., 2014b; Rose et al., 2015a). This adds a further level of realism to the trainee experience and offers new opportunities for educators in the anatomical and surgical sciences.

## 3D printing applications in anatomical modeling for teaching purposes

3D printing is able to generate accurate, tangible reproductions of anatomical structures, with faithful representations of both normal and pathologic variations (McMenamin et al., 2014; Vaccarezza and Papa, 2014; AbouHashem et al., 2015; Fredieu et al., 2015). This can be achieved in a relatively short period of time, at a relatively low cost and in numbers, which are suitable for manufacturers and educators alike (Torres et al., 2010; McMenamin et al., 2014; Vaccarezza and Papa, 2014; AbouHashem et al., 2015; Fredieu et al., 2015). While the accuracy of 3D prints is strongly dependent on the equipment used, it has been shown that 3D prints of anatomical structures could be produced with high accuracy compared to the original specimens (Li et al., 2012; McMenamin et al., 2014; Adams et al., 2015; Fredieu et al., 2015). A recent study reported a mean absolute error of 0.32 mm (variance 0.054 mm) for structures >10 mm in size (McMenamin et al., 2014). The prints can also be scaled up or down in overall size to suit the requirements of the educator, with accurate representation of colors and negative spaces as well (McMenamin et al., 2014). Additionally, the functionality of 3D printing and other rapid prototyping techniques allows for the production of different constituents of a specimen (such as bone, tendon, etc.) with different strength materials, thereby more accurately replicating the original (Waran et al., 2014b; Rose et al., 2015a). It was also suggested that 3D printing could be efficiently combined with more traditional techniques of anatomical modeling (O'Reilly et al., 2016).

3D printing appears to be particularly easy to implement in producing bone models (AbouHashem et al., 2015). Indeed, dry bones, being mainly monochromatic and made of hard tissue, seem to lend themselves naturally to printing. Both the shape and weight of a real bone could be copied with a high level of accuracy, preserving the haptic value, which is of vital importance in anatomy education (AbouHashem et al., 2015). The accuracy was demonstrated even in irregular bones, such as vertebrae (Ogden et al., 2014; AbouHashem et al., 2015). The only potential problem encountered was time needed to print individual bony element. For example, printing of a lumbar vertebra on a consumer grade 3D printer (MakerBot Replicator 2) took ~2 h (Ogden et al., 2014). At the same time the cost of material (PLA filament) of thus produced model is quite low—less than one American dollar.

Unsurprisingly, 3D prints have the potential to be a useful teaching tool for both anatomy students and patients. Preece et al. have shown improvement in veterinary students' anatomical test scores after use of 3D printing to teach equine limb anatomy (Preece et al., 2013). Patient understanding and consent of planned surgical procedures is also improved, as demonstrated in a recent study involving 3D printing of a lumbar spine model in anticipation of posterior lumbar fixation surgery (Liew et al., 2015). University level dental education offers another striking example of the utility of rapid prototyping technology for teaching purposes. Stereolithography techniques facilitated the cost-effective production of dental teaching blocks, with varying numbers of tooth cavities for student practice (Chan et al., 2004).

Studies investigating efficiency of 3D prints as teaching tools, as compared to traditional educational resources, particularly prosected human cadavers, have also started to appear. A recent pilot randomized control trial compared the performance in anatomy tests focusing on cardiac anatomy of three groups of students: those who learned 3D printed materials, those who learned from cadavers and those who learned from the combination of two (Lim et al., 2016). The study suggested that reliance on 3D prints did not disadvantages students in learning anatomy. 3D printed models also provide solutions to some of the difficulties faced by educators using more traditional methods of instruction.

3D printing techniques offer advantages over existing methods of anatomical modeling, which include cadaveric dissection, plastic models, and plastinated cadaver specimens (McMenamin et al., 2014). Anatomical tuition has traditionally been delivered primarily through cadaveric dissection; however this approach has been associated with a number of difficulties for educators. These include the cost involved in setting up and maintaining a dissection laboratory, sourcing sufficient numbers of cadavers through bequest programs, safety concerns for students and staff, and ethical concerns with using cadaveric material in some countries (McMenamin et al., 2014). Plastic models are also used as an adjunct to cadaveric dissection to demonstrate specific organ or skeletal anatomy. However, in the context of university and post-graduate curricula, they are often limited by their lack of anatomic realism and lack of representation of patient-specific variation or pathology (McMenamin et al., 2014). Plastination also suffers from drawbacks, primarily in regards to the resources required to create anatomical models.

Plastination is a relatively resource intensive technique, which involves infiltration of a dehydrated cadaver with a synthetic compound (McMenamin et al., 2014; Riederer, 2014). This process requires acquisition of sufficient numbers of cadaver material, manpower to produce the desired prosections, storage and then use of synthetic materials to create the final product (McMenamin et al., 2014; Riederer, 2014). In contrast, 3D printing can generate accurate reproductions, with less manpower and more control over the end product. Moreover, unlike plastination, the number of original specimens does not limit the quantity of 3D prints that can be produced.

Beyond the spectrum of anatomical modeling, 3D printing also has important applications in the field of surgery. These include applications for both surgical training, which aim to improve the experience of trainees, and also for surgical practice. The latter includes applications tailored to assist in a variety of areas, including pre-operative planning, simulation, execution, and implant/prosthetic production.

## 3D printing in surgical training

Surgical training has traditionally been delivered through an apprenticeship model, whereby a trainee would perform steps within a surgical procedure under the supervision of an expert. As the trainee gained competence, he/she would progress to more complex steps and be afforded more autonomy. The effectiveness and efficiency of this model is affected by the ratio of supervisors to trainees, the number of cases and range of pathology presenting/referred to a particular surgical unit and subsequently managed by trainees, and access to simulation aids such as cadaver material (Waran et al., 2014c). 3D printing technology offers solutions to bypass some of these issues and improve the trainee experience.

3D printing can produce accurate simulations of patient specific anatomy and pathology, which can then be used for pre-operative planning and skill acquisition. These models are based on real patient data, are reproducible, represent actual pathology and human variation, and are constructed with multiple materials designed to replicate real human tissue (McMenamin et al., 2014; Waran et al., 2014b; Rose et al., 2015a). Trainees can practice and master individual operative steps on the models prior to practice on a real patient. This can improve confidence amongst trainees (particularly in cases involving challenging or unusual anatomy) and help to accelerate the training timeline, as skill acquisition is obtained concurrently to real patient operative experience (Waran et al., 2014b,c; Rose et al., 2015b). Examples of challenging or unusual anatomy include cerebral arteriovenous malformations and aneurysms, other pathologies less frequently encountered in daily practice, and techniques such as ventricular endoscopy, which involve visualization constraints (Waran et al., 2015). It also helps to circumvent rate-limiting steps in the existing apprenticeship model of training, which include the need to balance patient safety with trainee operative practice, and dilution of operative exposure secondary to rising numbers of trainees (Waran et al., 2014c). Use of 3D printed models in training, also helps surgical educators to standardize operative skill acquisition amongst trainees (Waran et al., 2014b,c).

3D printing offers a number of advantages, which allow it to be a useful adjunct to existing methods of surgical simulation such cadaveric dissection and virtual reality techniques. Cadaver models have long been regarded as a gold standard for developing procedure specific surgical skills, due to its incorporation of real human anatomy and tissue handling characteristics (Blaschko et al., 2007). Cadaver based training remains highly effective and well regarded by surgical trainees and is likely to remain as a preferred training tool (Blaschko et al., 2007; Chambers et al., 2015). Its effectiveness has been demonstrated in a number of settings, including laparoscopic robot assisted surgery and orthopedic arthroplasty procedures (Blaschko et al., 2007; Chambers et al., 2015). However, as previously discussed, its use is limited by issues relating to cost, reproducibility and procurement (Waran et al., 2014c). Its effectiveness is also limited by the quality of cadaver tissue preservation and the degree of prior use, which can distort anatomical features (Blaschko et al., 2007). Virtual reality techniques, whilst becoming increasingly prominent in surgical training, also suffer from drawbacks. These include being devoid of necessary haptic feedback, and insufficient incorporation of anatomic realism and dynamic accuracy (accurate representation of organ/tissue behavior when physically handled; de Visser et al., 2011; Waran et al., 2014c).

In addition to surgical training, 3D printing has also yielded applications within surgical practice. This has been evident across a number of surgical specialties, including neurosurgery. Neurosurgical applications have included the production of models for the purposes of surgical planning and procedure simulation, and also the development of customizable patient specific implants and prosthetic devices.

## Applications of 3D printing in neurosurgery

**Figure 1:**
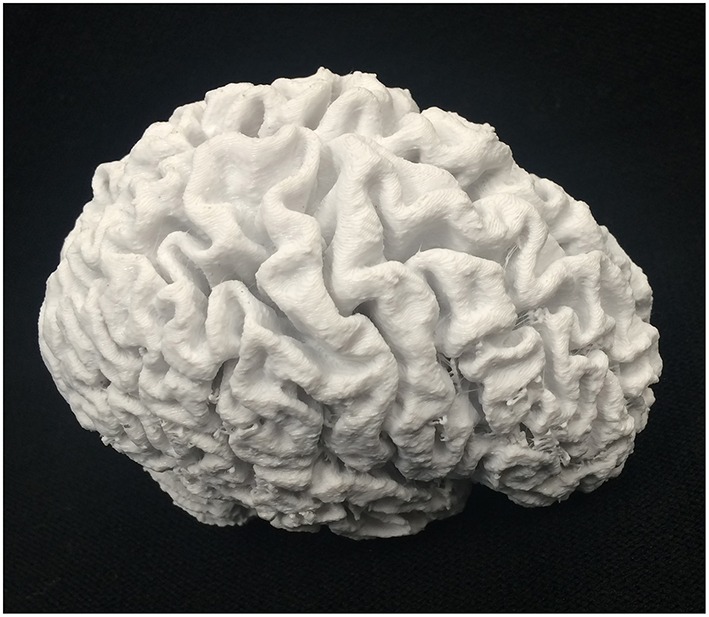
**Brain 3D model, printed from an MRI image on the MakerBot Replicator 2 3D printer**.

**Figure 2:**
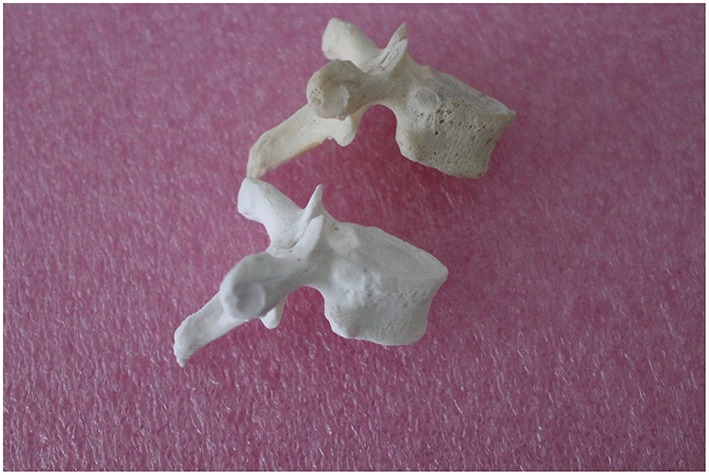
**Human thoracic vertebra (above) and 3D print of the same vertebra (below), made by the MakerBot Replicator 2**.

There have been a number of innovative applications of 3D printing within the context of neurosurgical training and operative planning. Neurosurgical anatomy is often exquisitely complex and cannot always be sufficiently appreciated via 2-dimensional multi-planar imaging (Klein et al., 2013). Accurate 3D models of patient specific anatomy enable the operator to visualize anatomical structures from different angles and also facilitate procedural skills practice. The latter is particularly important in neurosurgery, due to the high stakes nature of procedures within this specialty, and the otherwise slow step-wise accumulation of operative skill when working with real patients (Klein et al., 2013; Waran et al., 2015). A number of unique training models utilizing 3D printing techniques have emerged. See examples in Figures 1, 2.

Neurosurgical training models employing 3D printing have encompassed a number of common neurosurgical procedures and pathologies. There have been several proposed models for skill acquisition and operative planning for cerebrovascular disease, including aneurysm repair. Rapid prototyping technology, including 3D printing techniques, have been used to produce patient specific three dimensional cerebral aneurysm models, which can be used for pre-operative simulation of clipping repair (Wurm et al., 2004; Kono et al., 2013; Khan et al., 2014; Mashiko et al., 2015b). These have been constructed with silicone or rubber based materials, or photosensitive resin and similar models have also been successfully used to optimally shape microcatheters for intracranial aneurysm coiling (Wurm et al., 2004; Kono et al., 2013; Khan et al., 2014; Mashiko et al., 2015b; Namba et al., 2015). Pre-operative utilization of 3D printed AVM and vein of Galen models in children have been associated with shortened operative times (Weinstock et al., 2015).

Beyond the spectrum of cerebrovascular disease, 3D printing applications have also been developed for other parts of neurosurgical practice. Excision of an intracerebral lesion has been simulated via a multimaterial three-dimensional skull model, constructed with materials designed to replicate real tissue (Waran et al., 2014b,c). This can be used to practice operative steps, including creation of a skin flap, craniotomy, and lesion excision (Waran et al., 2014b,c). A similar multimaterial model, this time fluid filled and representing a patient with hydrocephalus secondary to pineal tumor, has been developed to aid in neuroendoscopic training and simulation of endoscopic third ventriculostomy and pineal biopsy (Waran et al., 2015). Other common neurosurgical operative steps, such as brain retraction and external ventricular drain placement have been simulated using rapid prototyped skull models, which have used multiple materials and patient specific source data to create a realistic training experience (Mashiko et al., 2015a; Tai et al., 2015). An additional application of rapid prototyped models within the context of surgical simulation is their ability to be incorporated into computer based navigation systems (Waran et al., 2012, 2014a, 2015). This can facilitate training of neurosurgical trainees in the use of these navigation systems, which are becoming more prevalent in neurosurgery (Waran et al., 2012, 2014a).

In addition to neurosurgical simulation and training, 3D printing has also been applied to the planning and execution of procedures. As discussed earlier, 3D printed models have been demonstrated to have value in the pre-operative assessment of pathology, such as aneurysms. They can also be used in the planning and preparation for complex and uncommon surgeries and can lead to reduced intra-operative errors and operating time (Muller et al., 2003). Additionally, they have been shown to improve patient understanding and informed consent before procedures (Liew et al., 2015). Apart from pre-operative planning, 3D printing has also been demonstrated to have a role in the actual performance of intra-operative steps. Patient specific 3D printed/rapid prototyped spinal laminar templates have been shown to be an effective intra-operative guide for pedicle screw fixation, thereby ensuring accuracy and reducing operating time in spinal fixation surgery (Lu et al., 2009; Sugawara et al., 2013). Similar outcomes have been achieved with the creation of customizable cranial implants.

Rapid prototyping technology has a role in the production of customizable cranial implants for patients undergoing cranioplasty. Several groups have shown improvements in morphology and aesthetic appearance using implants, which have been designed using CT data of an existing cranial defect and then produced using rapid prototyping techniques (Rotaru et al., 2006, 2012; Klammert et al., 2010; Esses et al., 2011; Chrzan et al., 2012; Tan et al., 2015). Materials used to create the implants include biocompatible materials such as polymethylmethacrylate (PMMA) and knitted polypropylene polyester (Rotaru et al., 2006, 2012; Chrzan et al., 2012). Traditionally, cranioplasty implants were adjusted and fitted intra-operatively (Chrzan et al., 2012). The use of customized implants facilitates faster operating times and assists with achieving better implant fits, particularly in complex cases (Chrzan et al., 2012).

## Pros and cons of 3D printing technology

As outlined above, 3D printing and other rapid prototyping technologies have a number of advantages over existing manufacturing techniques. They are able to produce customizable three-dimensional structures to a high level of accuracy. This can be done economically for small production runs, akin to mass production (Berman, 2012). As opposed to conventional manufacturing processes, rapid prototyping is entirely automated and uses readily available materials, thereby avoiding the need for supply chain integration (Berman, 2012). It also produces less waste and there is no unsold/unused inventory (Berman, 2012). Alongside its advantages, rapid prototyping also has a number of limitations.

The limitations of 3D printing and the other forms of rapid prototyping include the restriction of material choice to substances amenable to additive fabrication, which can limit choice of color and product durability/strength (Berman, 2012). The additive layering process can compromise surface finish and also pose difficulties for the creation of a working machine, whereby constituent parts work together to achieve a particular function. The size of objects produced is also limited by the size of the 3D printer, which can prevent large structures such as whole body models from being produced (Rengier et al., 2010). The cost is an additional limitation, with the rapid prototyping machines costing in the tens to hundreds of thousands of dollars, not including the cost of the plastic and resin based materials (Berman, 2012). It can also take hours to days to produce a final product, depending on the machine used and the complexity of the product (Berman, 2012). This may be justifiable for unique and complex applications but may be ill suited to more common scenarios or emergency cases (Rengier et al., 2010).

## Future perspectives

3D printing has opened up new and exciting avenues within the fields of anatomical education and modeling, surgical training and also within the discipline of neurosurgery. Applications in the latter have ranged from education and training, to assistance in daily surgical practice. It is likely that future developments will include a wider range of materials leading to more durable and realistic products. Improvements in the layering process, including fusion of substrate materials, are required to achieve greater product strength and precision in order to compete with more established manufacturing techniques (Berman, 2012). It is also likely that the cost and speed of 3D printers will improve, thereby increasing the usability and potentially the uptake of these machines (Berman, 2012). This has the potential to further revolutionize the anatomical and surgical sciences to the benefit of educators, surgeons, and patients.

## Author contributions

VB: article drafting, literature review. GS: supervision, figures, article editing. MS: supervision, article editing. ADI: project finalization, supervision, drafting, article editing.

## Funding

We would like to thank the Faculty of Science and Engineering at Macquarie University (Sydney, Australia) for the financial support provided in submitting this paper for publication.

### Conflict of interest statement

The authors declare that the research was conducted in the absence of any commercial or financial relationships that could be construed as a potential conflict of interest.
